# Positive affect training to reduce mental health problems during the COVID-19 pandemic: a proof-of-concept randomised clinical trial

**DOI:** 10.1136/bmjment-2023-300737

**Published:** 2023-06-28

**Authors:** Richard Bryant, Katie Dawson, Suzanna Azevedo, Srishti Yadav, Jenny Tran, Jasmine Choi-Christou, Elpiniki Andrew, Joanne Beames, Dharani Keyan

**Affiliations:** School of Psychology, University of New South Wales, Sydney, New South Wales, Australia

**Keywords:** depression & mood disorders, anxiety disorders, suicide & self-harm, COVID-19

## Abstract

**Background:**

The social restrictions occurring during the pandemic contributed to loss of many sources of reward, which contributes to poor mental health.

**Objective:**

This trial evaluated a brief positive affect training programme to reduce anxiety, depression and suicidality during the pandemic.

**Methods:**

In this single-blind, parallel, randomised controlled trial, adults who screened positive for COVID-19-related psychological distress across Australia were randomly allocated to either a 6-session group-based programme based on positive affect training (n=87) or enhanced usual care (EUC, n=87). Primary outcome was total score on the Hospital Anxiety and Depression Scale—anxiety and depression subscales assessed at baseline, 1-week post-treatment, 3 months (primary outcome time point) as well as secondary outcome measures of suicidality, generalised anxiety disorder, sleep impairment, positive and negative mood and COVID-19-related stress.

**Findings:**

Between 20 September 2020 and 16 September 2021, 174 participants were enrolled into the trial. Relative to EUC, at 3-month follow-up the intervention led to greater reduction on depression (mean difference 1.2 (95% CI 0.4 to 1.9)), p=0.003), with a moderate effect size (0.5 (95% CI 0.2 to 0.9)). There were also greater reduction of suicidality and improvement in quality of life. There were no differences in anxiety, generalised anxiety, anhedonia, sleep impairment, positive or negative mood or COVID-19 concerns.

**Conclusions:**

This intervention was able to reduce depression and suicidality during adverse experiences when rewarding events were diminished, such as pandemics.

**Clinical implications:**

Strategies to improve positive affect may be useful to reduce mental health issues.

**Trial registration number:**

ACTRN12620000811909.

**WHAT IS ALREADY KNOWN ON THIS TOPIC**

- Positive affect training aims to reduce anhedonia by promoting awareness of positive affect.
- Positive affect training delivered over 16 sessions has been shown to reduce anxiety and depression in randomised controlled trials.

**WHAT THIS STUDY ADDS**

- This trial demonstrated that a group-based 6-session positive affect training programme delivered via videoconferencing and targeted towards people distressed by the COVID-19 pandemic reduced depression, suicidality and improved quality of life relative to self-help.
- Positive affect training also resulted in less depression and suicidality, and improved quality of life in participants who initially had an anxiety or depression disorder.

**HOW THIS STUDY MIGHT AFFECT RESEARCH, PRACTICE OR POLICY**

- This programme offers the potential for health services during pandemics to alleviate increased mental health problems in a framework that is scalable to large numbers of people, even when there is social distancing.

## Introduction

The COVID-19 pandemic has caused marked increases in anxiety and depression around the world.^1^ Suicidal ideation has also been reported at high rates, with pooled estimates in the general population ranging from 11% to 12% (95% CI 9.3 to 15.2).^2^ Although there is evidence of increases in suicidal ideation and emergency department presentations due to suicidality,^3 4^ evidence does not suggest this has translated to increased suicide rates.^5^ Furthermore, longitudinal studies indicate these problems are not abating over the course of the pandemic.^6^ This increase in mental health problems can be attributed to a variety of factors, including lockdowns, social isolation, economic pressures and fear of infection.^7^

The rise in mental health needs globally has led to the need for psychological interventions that can be delivered during the pandemic. In many countries affected by the pandemic, there is a shortage of mental health services, many people cannot access mainstream services because of lockdowns or fear of being infected or many people are experiencing mental health problems for the first time and so are not accustomed to seeking mainstream mental health services.^8^ One of the major developments during the pandemic was for mental health delivery to occur via videoconferencing or telehealth formats.^9^ Several trials have demonstrated that videoconferencing or telehealth programmes based on cognitive behavioural approaches can be beneficial in reducing a range of common mental health problems observed during the pandemic, including loneliness, anxiety and depression.^10 11^ Overall, the majority of trials conducted for common mental disorders during the pandemic have been limited by small sample sizes or short follow-up periods. Furthermore, only one study has measured suicidal risk as an outcome and to date there is no evidence of suicidal risk reduction in these trials.^12^

One therapeutic approach that has not been evaluated for distress during the pandemic is positive affect training, which is a recent adaptation of cognitive behavioural techniques. This treatment differs from existing treatment packages because it focuses on strategies to enhance hedonic capacity that include positive event scheduling, imagination exercises to promote rewarding experiences and training savouring of the pleasurable aspects of positive experiences.^13^ Positive affect training extends beyond strategies such as behavioural activation because it teaches skills in recounting of positive experiences with explicit focus on optimising the experience of pleasure, and to use vivid imaginative techniques to plan the pleasure experienced derived from planned positive events.^14^ This intervention is predicated on the importance of promoting reward processes, and has been shown to be effective in reducing both negative affect and improving positive affect, as well as suicidal ideation.^15^ This approach may be particularly useful in the context of the pandemic because there is increasing recognition that anhedonia has been an ongoing problem throughout the pandemic,^16^ in part because social isolation and loneliness has deprived people of sources of reward and has contributed to anhedonia. Addressing anhedonia is important because during the pandemic anhedonia has been shown to have ripple effects on other psychological problems.^17^ Apart from its utility in reducing anhedonia, positive affect training has been shown to be a buffer against the effects of stressors.^18^

This trial evaluated a brief, transdiagnostic intervention provided during the pandemic that was an adaptation of positive affect training. Specifically, the current trial integrated positive affect training components into the programme and compared its efficacy relative to enhanced usual care (EUC). We hypothesised that the intervention would lead to reduce anxiety, depression and suicidality relative to EUC.

## Methods

### Trial design

This randomised, parallel, controlled trial randomly assigned distressed people during the COVID-19 pandemic to either the intervention or EUC on a 1:1 basis. The trial is reported in terms of the CONSORT guidelines.^19^ Participants were randomised by a researcher who was independent of the trial using computerised software to generate random number sequences with a block size of 4. The primary outcome time point was a 3-month follow-up after the intervention. All assessments were conducted online via a link emailed to participants by the trial coordinator, and in this way assessments were independent of any personnel involved in treatment condition allocation or delivery.

### Participants, recruitment and inclusion/exclusion criteria

We recruited participants through online advertising across Australia that informed potential participants of a trial to evaluate a programme to manage distress during the pandemic. Participants access a trial website, complete informed consent and then completed the Kessler Psychological Distress Scale (K10).^20^ Inclusion criteria included (a) adult (aged 18 years or older); (b) score ≥16 on the K10^21^ and (c) adequate English-language comprehension. Exclusion criteria were current psychosis, imminent suicidal risk, current substance dependence, current psychotherapy or no internet-based access for videoconferencing; these factors were assessed by self-report questions on the online screening platform. Any participants who endorsed suicidal plan or attempt in the past 6 months on dichotomously answered questions was excluded and referred to specialist services. Current psychotropic medication was permitted if the dosage was stable for 2 months.

### Interventions

The basic structure and content of the intervention was developed following focus groups with people affected by the pandemic. Before the group programme commenced, participants took part in an individual 15 min online session to explain the programme and ensure they understood the rules for the group participation. Brief prerecorded webinars were used in group discussions. Session 1 comprised education about common reactions to COVID-19, including anxiety, depression, worries, loss of pleasure and focused on strategies to heighten awareness of ways to savour positive experiences participants can have. The latter strategy instructed participants to use their imagination to rehearse and focus on positive elements of activities that they do engage in. Session 2 reviewed participants’ experiences of savouring events since the previous session, and instructed participants on how to recount prior positive experiences to elevate their mood. This recounting involved heightening awareness of affective, sensory and cognitive aspects of the experience. Participants were also assisted to identify meaningful activities that they could engage in, despite the limitations of lockdown. They were encouraged to identify and engaged in at least one activity per day, and to rehearse the savouring exercise with each activity. Participants were also instructed on the nature of rumination and worry during the pandemic, and the stress this way of thinking can cause. Participants were taught to discriminate between controllable and uncontrollable worries, and to engage in simple mindfulness exercises to not focus on uncontrollable worries. Slow breathing exercises were taught to augment this approach. Sessions 3 through 5 continued to review the activities previously taught, with a focus on trouble-shooting on how to savour activities and structure meaningful activities. Session 6 focused on reviewing the strategies and relapse prevention (for full programme, online supplemental material 1).

10.1136/bmjment-2023-300737.supp1 Supplementary data

Participants in the EUC condition were emailed handouts that contained the same education and coping strategies provided in the intervention arm, however participants in EUC were asked to rehearse these strategies in a self-paced manner over 6 weeks. We adopted this form of comparator because self-help coping strategies have been a very common mental health outreach initiative during the pandemic.

Sessions were audiorecorded and an independent psychologist rated 20% of sessions for treatment fidelity using a prescribed checklist. There was a high level of treatment fidelity, with 88% of rated sessions reflecting perfect adherence to the protocol. Treatment quality was also rated highly with a mean rating of 6.7 (possible range: 0–7). Adverse reactions, which were monitored each session by group facilitators and also on the basis of scheduled assessments, were referred to the Data Safety Monitoring Committee.

### Measures

All participants received an email with a link to an online assessment battery at baseline, postintervention and 3 months follow-up. None of the research team had access to these data prior to the final analysis, ensuring independence of assessments.

#### Primary outcome

We assessed the primary outcomes of anxiety and depressive symptoms with the Hospital Anxiety and Depression Scale (HADS^22^). The HADS was employed as a multiple primary outcome measure because it comprises a 14-item scale that has 2 subscales: HADS-A (anxiety, 7 items, range 0–21) and HADS-D (depression, 7 items, range 0–21). Higher scores on each subscale indicate more anxiety and depression, and has been shown to detect change in treatment trials.^23^ A subscale score ≥8 indicates probable caseness of anxiety and depression, respectively, and scores ≥15 indicate severe levels of each condition.^23^ We previously validated these self-reports of anxiety and depression on the HADS by having clinicians interview patients and estimate HADS scores based on interview responses, which yielded reliability between self-reported HADS and interview-based scores of 0.82 for depression and 0.88 for anxiety.^11^ In recognition of the multiple primary outcomes, we employed a Bonferroni adjusted alpha of 0.025 to determine statistical significance.

#### Secondary outcomes

Generalised anxiety and worry were assessed with the Generalised Anxiety Disorder Scale (GAD-7^24^), which is a 7-item self-report measure that possesses good psychometric properties, identifies people with severe worry, and is sensitive to change.^24^ A cut-off score of 15 has been recommended to indicate generalised anxiety disorder.^24^ Suicidal ideation was assessed with the Suicidal Ideation Attributes Scale (SIDAS^24^), which is a 5-item questionnaire on which each item is scored on 11-point scale, providing a range of 0–50. The SIDAS has strong internal consistency, convergent validity with measures of depression, a score ≥1 has good sensitivity for suicidal plans.^25^ Positive and negative affect was assessed with the Positive and Negative Affect Schedule (PANAS^26^) on which participants describe their mood by rating 10 positive and 10 negative words. Problems with sleep were assessed using an adapted version of the Sleep Impairment Index (SII^26^), which is 5-item measure of problems in sleep onset, maintenance, early waking, disturbance caused by sleep problems. The SII has good psychometric properties, is sensitive to treatment outcomes,^26^ and insomnia is indicated by a score ≥10.^27^ To assess pandemic-related worries, we adapted items from existing measure for a COVID Concerns Scale, which comprised nine items that were each scored on a 5-point scale (0=*not at all*, 4=*extremely*), with higher scores indicating greater concerns; this scale had adequate internal consistency in the current sample (Cronbach’s alpha=0.77). Quality of life was assessed with the Australian Quality of Life Scale (AQoL-8D^28^), which measures quality of life and health outcomes across eight domains (independent living, relationships, mental health, coping, pain, senses, self-worth and happiness).

### Statistical analyses

The sample size was calculated on the basis of prior trials of group-based interventions delivered during the pandemic,^8^ which indicated that to achieve an effect size of (0.3) a sample size of 67 participants would be needed per arm to provide power of 0.95 (alpha=0.05, two-sided); on the expectation that there would be approximately 30% attrition at the 3-month follow-up assessment, we calculated that a total of 174 participants would be required for the study.

We assessed potential differences between participants across treatment arms using planned t-tests for continuous measures and χ^2^ statistics for categorical variables. The primary analyses employed an intent-to-treat approach in which hierarchical linear models were used to study the effects of each treatment condition. This statistical approach allows the number of observations to vary between participants, which handles missing data by calculating estimates of trajectories using maximum likelihood estimation. Fixed effects were tested for intervention condition and time of assessment, with parameters tested using the Wald test (t-test, p<0.05, two-sided) and 95% CIs. Analyses focus on the primary (HADS) and secondary (GAD-7, SII, PANAS, SIDAS, AQoL-8D and COVID Concerns Scale) outcomes, with the main outcome timepoint being the 3-month follow-up. Effect sizes were calculated by dividing the estimated mean difference between treatment arms by the baseline pooled SD. We also calculated minimally important differences for the primary outcomes of the HADS anxiety and depression scales by using the recommended cut-offs of 1.32 for the HADS-A scale and 1.40 for the HADS-D scale.^29^ ^30^ We conducted secondary analyses to assess if our statistical approach was biased by attrition at the 3-month follow-up by repeating the analyses using only participants who completed the 3-month follow-up. Furthermore, we also assessed the effect of the treatment when we restricted the analyses to participants with more severe psychological disorders, as operationalised the recommended cut-offs for severe anxiety or depression on the HADS anxiety and depression subscales, respectively^24^ or for probable generalised anxiety disorder on the GAD-7.^25^

## Results

### Baseline characteristics of participants

**Figure 1:**
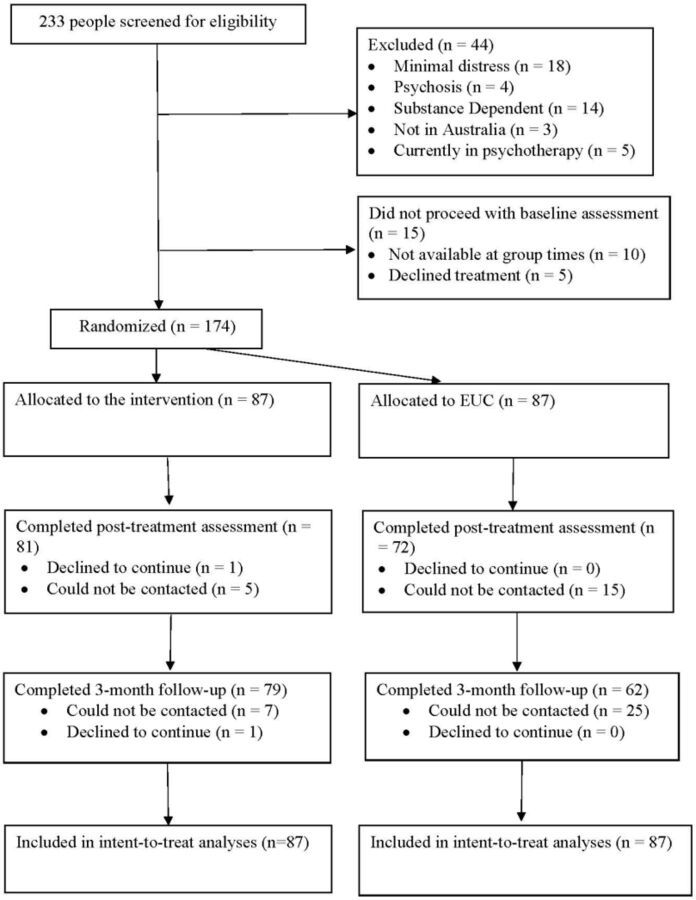
Study flow diagram. EUC, enhanced usual care.

Between 10 December 2020 and 16 September 2021 (with final 3-month follow-up assessment completed on 18 February 2022), 174 participants were enrolled into the trial. The majority of participants were female (83.9%), and were university educated (71.3%). In terms of caseness of psychological problems, 85.1% met the cut-off for probable depression (2.3% for severe depression), 79.3% for probable anxiety disorder (1.7% for severe anxiety), 32.0% for generalised anxiety disorder. There were 87 participants randomised to the intervention and 87 to EUC. Participants in the intervention and EUC did not differ at baseline on any sociodemographic characteristics or baseline psychopathology measures (table 1). The primary outcome assessment at 3 months was conducted for 79 (90.8%) participants in the intervention and 62 (71.3%) in EUC. The flow chart of participant recruitment and retention is reported in figure 1. Participants who were and were not retained at the 3-month follow-up did not differ on baseline demographic characteristics; those who were not retained had lower levels of sleep impairment and suicidality (online supplemental table S1).

**Table 1:** Demographic and participant characteristics

	Intervention (n=87)	Enhanced usual care (n=87)
Age, years	36.1±11.6	351±11.0
Female sex, no. (%)		
Female	71 (81.6)	75 (86.2)
Male	13 (14.9)	10 (11.5)
Non-binary	3 (3.4)	2 (2.3)
Employment status, no. (%)		
Full-time	31 (35.6)	38 (43.7)
Part-time	23 (26.4)	20 (23.0)
Student	11 (12.6)	12 (13.8)
Unemployed	15 (17.2)	10 (11.5)
Retired	7 (8.0)	7 (8.0)
Relationship status, no. (%)		
Spouse	42 (48.3)	28 (32.2)
Divorced/Separated	5 (5.7)	12 (13.8)
Widowed	0 (0.0)	1 (1.1)
Single	40 (46.0)	46 (52.9)
Education, no. (%)		
High school	10 (11.5)	11 (12.6)
Trade certificate	9 (10.3)	7 (8.0)
Diploma	6 (6.9)	7 (8.0)
Bachelor’s degree	31 (35.6)	40 (46.0)
Higher degree	31 (35.6)	22 (25.3)
Ethnicity, no. (%)		
Australian	57 (65.5)	57 (65.5)
Asian	14 (2.3)	9 (10.3)
European	8 (9.2)	12 (13.8)
Middle Eastern	2 (2.3)	3 (3.4)
Indigenous Australian	0 (0.0)	2 (2.3)
Other	6 (6.9)	4 (4.6)
Moderate-to-severe depression, no. (%)	77 (81.6)	71 (81.6)
Moderate-to-severe anxiety, no. (%)	69 (79.3)	69 (79.3)
Generalised anxiety disorder, no. (%)	67 (77.0)	68 (78.2)
Insomnia, no. (%)	74 (85.0)	70 (80.5)

Moderate/Severe depression=HADS-Depression Scale score ≥15. Moderate/Severe anxiety=HADS-Anxiety Scale score ≥15. Generalised anxiety disorder=GAD-7 score ≥15. Insomnia=Sleep Impairment Index score ≥10.

GAD-7, Generalised Anxiety Disorder Scale; HADS, Hospital Anxiety and Depression Scale.

### Primary outcome

At 3 months participants in the intervention arm reported greater reductions in depression on the HADS (mean difference 1.2 (95% CI 0.4 to 1.9), p=0.003), with a moderate effect size (0.5 (95% CI 0.2 to 0.9)). There was no between-group difference on anxiety on the HADS (table 2). There were more participants in the intervention arm (34; 43.0%) relative to EUC (16; 25.8%) achieving a minimally important difference in depression between baseline and follow-up (χ^2^=4.5, p=0.03). There was no difference in proportions of participants achieving a minimally important difference between the intervention (19; 24.1%) and EUC (21; 33.9%) arms for anxiety (χ^2^=1.6, p=0.20).

**Table 2:** Summary statistics and results from mixed model analysis of primary and secondary outcomes

Primary and secondary outcomes	Visit	Descriptive statistics		Mixed model analysis		
		Intervention (n=87)	EUC (n=87)	Difference in LS mean (95% CI)	P value	Effect size (95% CI)
		Estimated mean (95% CI)	Estimated mean (95% CI)			
HADS-depression	Baseline	10.0 (9.5 to 10.4)	9.1 (8.6 to 19.5)			
	7 weeks	8.9 (8.4 to 9.3)	8.8 (18.4 to 9.2)	0.8 (0.1 to 1.6)	0.03	0.4 (0.0 to 0.7)
	3 months	8.8 (8.5 to 9.3)	9.0 (9.6 to 9.5)	1.2 (0.4 to 1.9)	0.003	0.5 (0.2 to 0.9)
HADS-anxiety	Baseline	9.2 (8.6 to 9.8)	9.9 (9.4 to 10.5)			
	7 weeks	10.9 (10.4 to 11.5)	10.9 (10.3 to 11.4)	−0.9 (−1.8 to 0.0)	0.06	−0.3 (−0.7 to 0.0)
	3 months	10.2 (9.6 to 10.8)	10.5 (9.8 to 11.1)	−0.5 (−1.5 to 0.4)	0.25	−0.2 (−0.6 to 0.1)
Suicidality	Baseline	6.6 (4.3 to 8.9)	3.1 (4.3 to 8.8)			
	7 weeks	3.3 (0.9 to 5.7)	4.1 (1.6 to 6.7)	4.3 (0.6 to 8.0)	0.03	0.4 (0.1 to 0.7)
	3 months	2.9 (0.6 to 5.2)	4.4 (1.9 to 6.9)	5.0 (1.5 to 8.5)	0.006	0.4 (0.1 to 0.7)
GAD	Baseline	12.3 (11.3 to 13.4)	11.3 (10.2 to 12.3)			
	7 weeks	7.1 (6.2 to 8.1)	7.6 (6.5 to 8.6)	1.5 (0.0 to 2.7)	0.05	0.3 (0.0 to 0.6)
	3 months	7.3 (6.2 to 8.4)	7.6 (6.4 to 8.7)	1.3 (−0.2 to 2.9)	0.10	0.3 (0.0 to 0.6)
PANAS-positive	Baseline	20.6 (18.9 to 22.2)	21.5 (19.9 to 23.1)			
	7 weeks	25.2 (23.3 to 27.5)	24.5 (22.3 to 26.8)	−1.7 (−4.9 to −1.4)	0.27	−0.2 (−0.6 to −0.2)
	3 months	25.0 (23.1 to 27.0)	24.1 (22.0 to 26.2)	−1.8 (−4.8 to 1.1)	0.21	−0.2 (−0.6 to 0.1)
PANAS-negative	Baseline	21.5 (19.7 to 23.3)	21.4 (19.6 to 23.2)			
	7 weeks	19.1 (17.3 to 20.9)	18.3 (16.4 to 20.2)	−0.7 (−3.7 to 2.3)	0.66	−0.1 (−0.4 to 0.3)
	3 months	19.7 (17.9 to 21.5)	19.2 (17.3 to 21.2)	−0.3 (−3.1 to 2.5)	0.84	0.0 (−0.4 to 0.3)
Quality of life	Baseline	47.1 (45.0 to 49.2)	45.1 (43.0 to 47.2)			
	7 weeks	41.8 (39.5 to 44.2)	41.6 (39.2 to 44.1)	1.8 (−1.2 to 4.7)	0.23	0.2 (−0.1 to 0.5)
	3 months	40.8 (38.5 to 43.4)	42.5 (39.9 to 45.1)	3.6 (0.3 to 6.9)	0.03	0.4 (0.0 to 0.7)
COVID Concerns Scale	Baseline	25.9 (24.4 to 27.3)	25.8 (24.3 to 27.2)			
	7 weeks	22.3 (20.6 to 24.1)	22.3 (20.5 to 24.1)	0.5 (−2.5 to 2.6)	0.97	0.1 (−0.3 to 0.4)
	3 months	22.2 (20.5 to 23.9)	22.7 (20.8 to 24.5)	0.6 (−2.0 to 3.2)	0.64	0.1 (−0.3 to 0.5)
Sleep disturbance	Baseline	9.2 (8.3 to 10.0)	8.7 (7.8 to 9.5)			
	7 weeks	6.5 (5.6 to 7.4)	6.9 (5.9 to 7.9)	0.9 (−0.4 to 2.2)	0.18	0.2 (−0.1 to 0.6)
	3 months	5.9 (4.9 to 6.9)	6.3 (5.2 to 7.4)	0.8 (−0.6 to 2.3)	0.26	0.2 (−0.2 to 0.6)

Effect size was calculated by the difference in least square means between intervention and EUC from mixed model divided by the pooled SD.

Sleep Impairment Index (total score range: 0–20; higher scores indicate more severe sleep impairment).

COVID Concerns Scale, COVID-19 Stress Scale (each scale total score range: 0–24; higher scores indicate more severe stress); EUC, enhanced usual care; GAD-7, Generalised Anxiety Disorder Scale (total score range: 0–21; higher scores indicate more severe worry); HADS, Hospital Anxiety and Depression Scale (depression subscale score range: 0–21; anxiety subscale score range: 0–21; higher scores indicate elevated anxiety or depression); LS, least square; PANAS, Positive and Negative Affect Schedule (subscale total score range: 10–50 on positive and negative scales, respectively; higher scores indicate more greater positive and negative mood, respectively); Quality of life, Australian Quality of Life Scale (total score range: 20–100; higher scores indicate poorer quality of life); Suicidality, Suicidal Ideation Attributes Scale (total score range: 0–50; higher scores indicate more severe suicidality).

### Secondary outcomes

In terms of suicidality, the intervention led to greater reduction difference at 3-month follow-up (mean difference 4.8 (95% CI 1.32 to 8.4), p=0.008), indicating a moderate effect size (0.4 (95% CI 0.1 to 0.8) (table 2). The intervention also led to greater improvement in quality of life (mean difference 3.5 (95% CI 0.2 to 6.9), p=0.04), indicating a moderate effect size (0.4 (95% CI 0.0 to 0.7)). There were no significant effects between treatment arms for the secondary outcomes of GAD, positive or negative affect, COVID-19 concerns or sleep disturbance.

### Subgroup analyses

There were 148 participants who reported probable depression or anxiety at baseline; these were equally distributed between the intervention (77, 88.5%) and EUC (71 (81.6%). In terms of analyses that focused on these participants (online supplemental table S2), the intervention resulted in greater reductions at 3-month follow-up on depression (mean difference 1.0 (95% CI 0.3 to 1.8), p=0.009), indicating a moderate effect size (0.6 (95% CI 0.2 to 1.0)). Furthermore, there were greater reductions in suicidality 6.2 (95% CI 2.3 to 10.2), p=0.002) and greater increases in quality of life (4.2 (95% CI 0.5 to 7.9), p=0.03) in the intervention arm relative to EUC.

### Sensitivity analyses

The sensitivity analysis that focused only on participants who completed the 3-month follow-up indicated that in terms of primary outcomes, the intervention led to greater reductions than EUC for depression and suicidality, and greater increase in quality of life (online supplemental table S3). This pattern of results mimics the results observed in the linear mixed models, and suggests that our intent-to-treat approach was robust.

There were no adverse events throughout the trial.

## Discussion

This study demonstrated for the first time that a brief behavioural programme specifically designed to address anhedonia in the context of the COVID-19 pandemic can reduce depression, suicidality and improve quality of life. Considering this is a brief 6-session group-based programme, the observed effects 3 months following the intervention are promising and suggest that this intervention has the potential for implementation in large-scale crises when people’s normal sources of pleasure are restricted, such as in pandemic-related lockdowns. The delivery of the programme via videoconferencing also suggests this intervention can be effective when delivered remotely, which is desirable in situations when physical proximity is not possible due to lockdowns or quarantine, or where distance prevents people from attending sessions face-to-face.

The finding that positive affect training reduced depression and suicidality is consistent with a previous trial that showed that positive affect training reduced negative mood and suicidality, and improved positive affect.^15^ Notably, this previous trial treated patients on an individual basis over 15 sessions. Although the current programme was much briefer, it shows that promoting reward processes in the context of diminished sources of pleasure during the pandemic can have important benefits of reducing negative emotional states. In the context of isolation and lockdowns during the pandemic contributing to anhedonia, the potential to boost anhedonic capacity by brief positive affect training is a useful addition to current psychological interventions. The focus on promoting positive affect is underscored by evidence that deficient capacity to experience pleasure is a predictor of worsening mental health conditions, such as depression and suicidality,^31^ ^32^ outcomes that were shown in the current trial to be reduced by the intervention.

We note that the intervention did not impact on anxiety, positive or negative mood, generalised anxiety, sleep disturbance or pandemic concerns. This pattern of findings contrasts with a previous trial of positive affect training that observed that this intervention significantly reduced anxiety.^15^ We note this trial provided 15 individual sessions to participants, and it is possible that the brevity and lack of individual attention contributed to the current intervention not achieving reductions in anxiety or related states. The absence of an effect on pandemic-related concerns is in contrast to an earlier group intervention that focused on problem management and reducing worries during the pandemic.^11^ Taken together, it appears that the current brief intervention that strives to improve positive affect can result in diminished depression and suicidality, as well as improved quality of life, but that its benefits are limited by not addressing other domains of psychopathology.

In terms of study limitations, most participants in the trial were female and had tertiary education. These results will need to be replicated with broader sampling to test the extent to which the findings are applicable to the general community. We also note that our primary outcome measure, the HADS, was conducted via self-report rather than structured clinical interview. Although we have previously shown that using the HADS in this format is comparable to clinical ratings of depression and anxiety,^11^ we recognise that this self-report does not represent the same level of assessment as structured clinical interview. We also note that considering that participants were aware of the treatment arm they were assigned to, it is possible that this form of assessment may not have been fully free of bias. The control condition involved participants following a self-help manual that comprised the same strategies that were taught in the positive affect training sessions. This design does not control for the non-specific effects of group involvement or attention of a group facilitator. Future trials could usefully employ more active control conditions that disentangle the non-specific from positive affect training components of the intervention. Finally, we note that this intervention was delivered by clinical psychologists, and if substantive scale-up is to be achieved of this intervention during pandemics or societal crises, especially in low-income and middle-income countries where there is a dearth of mental health specialists, there is a need for structured training protocols to be developed for people with varying qualifications.

In summary, this trial suggests that depression and suicidality can be reduced, and quality of life can be improved, with a brief group intervention that focuses on addressing anhedonia via positive affect training. In the context of social isolation and restricted access to traditional sources of reward, this intervention offers a potentially scalable intervention that could be useful during periods characterised by social restrictions or limited behavioural activities.

## Data Availability

Data are available on reasonable request. Data will be available for meta-analysis and other reasonable requests from RB (r.bryant@unsw.edu.au)
