# Supplementary material for: Positive affect training to reduce mental health problems during the COVID-19 pandemic: a proof-of-concept randomised clinical trial
Source: BMJ Ment Health. 2023 Jun 28;26(1):e300737. doi: 10.1136/bmjment-2023-300737 (PMC10577780; doi:10.1136/bmjment-2023-300737)
Supplement: Supplementary data [file bmjment-2023-300737supp001.pdf]

Supplemental material BMJ Publishing Group Limited (BMJ) disclaims all liability and responsibility arising from any reliance placed on this supplemental material which has been supplied by the author(s) BMJ Ment Health

CONSORT 2010 checklist of information to include when reporting a randomised trial*

Section/Topic	Item No	Checklist item	Reported on page No
**Title and abstract**			
	1a	Identification as a randomised trial in the title	1
	1b	Structured summary of trial design, methods, results, and conclusions (for specific guidance see CONSORT for abstracts)	2
**Introduction**			
Background and objectives	2a	Scientific background and explanation of rationale	4
	2b	Specific objectives or hypotheses	4
**Method**			
Trial design	3a	Description of trial design (such as parallel, factorial) including allocation ratio	5
	3b	Important changes to methods after trial commencement (such as eligibility criteria), with reasons	6
Participants	4a	Eligibility criteria for participants	5
	4b	Settings and locations where the data were collected	5
Interventions	5	The interventions for each group with sufficient details to allow replication, including how and when they were actually administered	6
Outcomes	6a	Completely defined pre-specified primary and secondary outcome measures, including how and when they were assessed	6-7
	6b	Any changes to trial outcomes after the trial commenced, with reasons	N/A
Sample size	7a	How sample size was determined	7
	7b	When applicable, explanation of any interim analyses and stopping guidelines	N/A
Randomisation:			
Sequence generation	8a	Method used to generate the random allocation sequence	4
	8b	Type of randomisation; details of any restriction (such as blocking and block size)	4
Allocation concealment mechanism	9	Mechanism used to implement the random allocation sequence (such as sequentially numbered containers), describing any steps taken to conceal the sequence until interventions were assigned	4
Implementation	10	Who generated the random allocation sequence, who enrolled participants, and who assigned participants to interventions	4
Blinding	11a	If done, who was blinded after assignment to interventions (for example, participants, care providers, those	4

de Graaff AM, *et al. BMJ Ment Health* 2023; 26:e300637. doi: 10.1136/bmjment-2022-300637

Bryant R, *et al. BMJ Ment Health* 2023; 26:e300737. doi: 10.1136/bmjment-2023-300737

Supplemental material BMJ Publishing Group Limited (BMJ) disclaims all liability and responsibility arising from any reliance placed on this supplemental material which has been supplied by the author(s) BMJ Ment Health

		assessing outcomes) and how	
	11b	If relevant, description of the similarity of interventions	N/A
Statistical methods	12a	Statistical methods used to compare groups for primary and secondary outcomes	7-8
	12b	Methods for additional analyses, such as subgroup analyses and adjusted analyses	7-8
**Results**			
Participant flow (a diagram is strongly recommended)	13a	For each group, the numbers of participants who were randomly assigned, received intended treatment, and were analysed for the primary outcome	8
	13b	For each group, losses and exclusions after randomisation, together with reasons	8
Recruitment	14a	Dates defining the periods of recruitment and follow-up	8
	14b	Why the trial ended or was stopped	8
Baseline data	15	A table showing baseline demographic and clinical characteristics for each group	8
Numbers analysed	16	For each group, number of participants (denominator) included in each analysis and whether the analysis was by original assigned groups	9-10
Outcomes and estimation	17a	For each primary and secondary outcome, results for each group, and the estimated effect size and its precision (such as 95% confidence interval)	9-10
	17b	For binary outcomes, presentation of both absolute and relative effect sizes is recommended	10
Ancillary analyses	18	Results of any other analyses performed, including subgroup analyses and adjusted analyses, distinguishing pre-specified from exploratory	9
Harms	19	All important harms or unintended effects in each group (for specific guidance see CONSORT for harms)	10
**Discussion**			
Limitations	20	Trial limitations, addressing sources of potential bias, imprecision, and, if relevant, multiplicity of analyses	11
Generalisability	21	Generalisability (external validity, applicability) of the trial findings	11-12
Interpretation	22	Interpretation consistent with results, balancing benefits and harms, and considering other relevant evidence	10-12
**Other information**			
Registration	23	Registration number and name of trial registry	5
Protocol	24	Where the full trial protocol can be accessed, if available	5
Funding	25	Sources of funding and other support (such as supply of drugs), role of funders	15

*We strongly recommend reading this statement in conjunction with the CONSORT 2010 Explanation and Elaboration for important clarifications on all the items. If relevant, we also recommend reading CONSORT extensions for cluster randomised trials, non-inferiority and equivalence trials, non-pharmacological treatments, herbal interventions, and pragmatic trials. Additional extensions are forthcoming: for those and for up to date references relevant to this checklist, see www.consort-statement.org.

Table S1. Participant characteristics of participants who were retained and lost at 3-months follow-up

	Retained (n = 141)	Not Retained (n = 33)	t/χ^2	*P*
Female, n (%)	119 (84.4)	27 (81.8)	1.5	0.47
Age, y (±SD)	36.5±12.8	38.3±13.0	0.9	0.4
Relationship status, n (%)			4.7	0.32
Spouse	57 (40.4)	13 (39.4)		
Single	71 (50.4)	15 (45.5		
Separated	13 98.2)	5 (15.1)		
Education			4.0	0.40
High School	17 (12.1)	4 (12.1)		
Diploma	11 (7.8)	2 (6.1)		
Trade certificate	10 (7.1)	6 (18.2)		
University	103 (73.0)	21 (63.6)		
Employment			1.1	0.90
Employed	88(63.1)	23 (69.7)		
Student	19 (13.5)	4 (12.1)		
Unemployed/retired	33 (23.4)	6 (18.2)		
Ethnicity			3.4	0.63
Australian	92 (65.2)	22 (66.7)		
Asian	20 (14.2)	3 (9.1)		
European	17 (12.1)	3 (9.1)		
Middle Eastern	3 (2.1)	2 (6.1)		

Indigenous	2 (1.4)	0 (0.0)		
Other	7 (13.7)	3 (10.3)		
HADS: Depression, *M* (±SD)	9.5±2.2	9.6±2.0	0.2	0.43
HADS: Anxiety, *M* (±SD)	9.4±2.6	10.2±3.2	1.5	0.12
GAD-7, *M* (±SD)	11.9±4.8	11.5±5.3	0.4	0.69
Sleep Impairment Index, *M* (±SD)	9.2±3.9	7.6±4.0	2.1	0.04
PANAS-Positive, *M* (±SD)	20.7±7.6	22.3±8.2	-1.0	0.30
PANAS-Negative, *M* (±SD)	21.5±8.4	21.3±9.0	0.2	0.87
Suicidality, *M* (±SD)	5.7±11.1	1.2±9.4	2.2	0.03
COVID-19 Concerns, *M* (±SD)	25.8±6.6	25.6±8.2	0.2	0.88
AQol, *M* (±SD)	46.6±10.0	43.7±9.3	1.5	0.14

Abbreviations. EUC = Enhanced usual care; LS = Least Square; HADS = Hospital Anxiety and Depression Scale (depression subscale score range: 0-21; anxiety subscale score range: 0-21; higher scores indicate elevated anxiety or depression); Suicidality = Suicidal Ideation Attributes Scale (total score range: 0-50; higher scores indicate more severe suicidality); GAD-7 (total score range: 0-21; higher scores indicate more severe worry); PANAS = Positive and Negative Affect Schedule (subscale total score range: 10-50 on positive and negative scales, respectively; higher scores indicate more greater positive and negative mood, respectively); Quality of Life = Australian Quality of Life scale (total score range: 20-100; higher scores indicate poorer quality of life); COVID Concerns = CSS = COVID-19 Stress Scale (each scale total score range: 0-24; higher scores indicate more severe stress); SII = Sleep Impairment Index (total score range: 0-20; higher scores indicate more severe sleep impairment). Effect size was calculated by the difference in least square means between intervention and EUC from mixed model divided by the pooled standard deviation.

Table S2. Summary statistics and results from mixed model analysis of primary and secondary outcomes for participants who completed the 6-month assessment

		Descriptive statistics		Mixed model analysis		
		Intervention (n = 79)	EUC (n = 62)			
Primary and secondary outcomes	Visit	Estimated Mean (95% CI)	Estimated Mean (95% CI)	Difference in LS mean (95%CI)	P-value	Effect size[a] (95% CI)
HADS-Depression	Baseline	10.0 (9.5 to 10.4)	9.1 (8.6 to 19.5)			
	7-week	8.9 (8.4 to 9.3)	8.8 (18.4 to 9.2)	0.8 (0.1 to 1.6)	.03	0.4 (0.0 to 0.7)
	3-month	8.8 (8.5 to 9.3)	9.0 (9.6 to 9.5)	1.2 (0.4 to 1.9)	.003	0.5 (0.2 to 0.9)
HADS-Anxiety	Baseline	9.1 (8.6 to 16.8)	9.7 (9.1 to 10.4)			
	7-week	10.9 (10. to 11.5)	10.7 (10.1 to 11.4)	-0.8 (-1.8 to 0.1)	.07	-0.8 (-1.8 to 0.1)
	3-month	10.2 (9.6 to 10.8)	10.3 (9.6.to 11.0)	-0.5 (-1.9 to- 0.9)	.31	-0.2 (-0.7 to 0.3)
Suicidality	Baseline	7.0 (0.9 to 6.0)	4.0 (1.3 to 6.8)			
	7-week	3.5 (0.9 to 6.0)	4.6 (1.7 to 7.4)	4.1 (0.0 to 8.1)	.048	0.4 (0.0 to 0.7)
	3-month	3.1 (0.7 to 5.5)	4.9 (2.2 to 7.5)	4.7 (1.0 to 8.5)	.01	0.4 (0.1 to 0.8)
GAD	Baseline	12.2 (11.1 to 13.3)	11.5 (10.2 to 12.7)			
	7-week	7.1 (6.1 to 8.1)	7.6 (6.5 to 8.7)	1.3 (-0.2 to 3.0)	.10	0.3 (0.0 to 0.6)
	3-month	7.2 (6.1 to 8.3)	7.6 (6.4 to 8.8)	1.2 (-0.4 to 2.8)	.15	0.2 (-0.1 to 0.6)
PANAS-Positive	Baseline	20.1 (18.4 to 21.8)	21.5 (19.6 to 23.4)			
	7-week	25.2 (23.0 to 27.3)	24.4 (21.9 to 26.8)	-2.2 (-5.5 to -1.1)	.19	-0.3 (-0.7 to -0.1)
	3-month	24.8 (22.8 to 26.8)	24.0 (21.8 to 26.3)	-2.2 (-5.2 to 7.9)	.15	-0.3 (-0.7 to 1.0)
PANAS-Negative	Baseline	21.1 (19.2 to 23.0)	22.1 (20.0 to 24.2)			
	7-week	18.8 (17.0 to 20.6)	18.4 (16.3 to 20.4)	-1.4 (-4.6 to 1.8)	.38	-0.2 (-0.5 to 0.2)
	3-month	19.4 (17.6 to 21.2)	19.4 (17.4 to 21.5)	-1.0 (-3.9 to 1.9)	.51	-0.1 (-0.5 to 0.2)
Quality of Life	Baseline	47.4 (45.1 to 49.6)	45.6 (43.1 to 48.2)			
	7-week	41.8 (39.3 to 44.3)	41.8 (39.0 to 44.6)	1.7 (-1.1 to 4.5)	.24	0.2 (-0.1 to 0.5)
	3-month	40.8 (38.3 to 43.3)	42.6 (39.8 to 45.5)	3.5 (0.2 to 6.8)	.04	0.4 (0.0 to 0.7)
COVID Concerns	Baseline	25.9 (24.4 to 27.4)	25.9 (24.1 to 27.5)			
	7-week	22.3 (20.5 to 24.0)	22.1 (20.2 to 24.1)	-0.1 (-2.6 to 2.5)	.95	0.0 (-04 to 0.4)
	3-month	22.1 (20.4 to 23.9)	22.6 (20.6 to 24.6)	0.5 (-0.2 to 3.2)	.69	0.1 (0.0 to 0.5)
Sleep Disturbance	Baseline	9.5 (8.6 to 10.3)	9.0 (7.9 to 9.9)			
	7-week	6.5 (5.5 to 7.4)	6.9 (5.8 to 7.9)	0.9 (-0.4 to 2.3)	.18	0.2 (-0.1 to 0.6)

		Descriptive statistics		Mixed model analysis		
		Intervention (n = 79)	EUC (n = 62)			
Primary and secondary outcomes	Visit	Estimated Mean (95% CI)	Estimated Mean (95% CI)	Difference in LS mean (95%CI)	P-value	Effect size[a] (95% CI)
	3-month	6.0 (5.0 to 7.0)	6.3 (5.2 to 7.4)	0.9 (-0.6 to 2.4)	.25	0.2 (-0.2 to 0.6)

Abbreviations. EUC = Enhanced usual care; LS = Least Square; HADS = Hospital Anxiety and Depression Scale (depression subscale score range: 0-21; anxiety subscale score range: 0-21; higher scores indicate elevated anxiety or depression); Suicidality = Suicidal Ideation Attributes Scale (total score range: 0-50; higher scores indicate more severe suicidality); GAD-7 (total score range: 0-21; higher scores indicate more severe worry); PANAS = Positive and Negative Affect Schedule (subscale total score range: 10-50 on positive and negative scales, respectively; higher scores indicate more greater positive and negative mood, respectively); Quality of Life = Australian Quality of Life scale (total score range: 20-100; higher scores indicate poorer quality of life); COVID Concerns = CSS = COVID-19 Stress Scale (each scale total score range: 0-24; higher scores indicate more severe stress); SII = Sleep Impairment Index (total score range: 0-20; higher scores indicate more severe sleep impairment). Effect size was calculated by the difference in least square means between intervention and EUC from mixed model divided by the pooled standard deviation. Effect size was calculated by the difference in least square means between intervention and EUC from mixed model divided by the pooled standard deviation.

Bryant R, *et al. BMJ Ment Health* 2023; 26:e300737. doi: 10.1136/bmjment-2023-300737

Table S3. Summary statistics and results from mixed model analysis of primary and secondary outcomes for participants with probable anxiety or depression at baseline

		Descriptive statistics		Mixed model analysis		
		Intervention (n = 85)	EUC (n = 85)			
Primary and secondary outcomes	Visit	Estimated Mean (95% CI)	Estimated Mean (95% CI)	Difference in LS mean (95%CI)	P-value	Effect size[a] (95% CI)
HADS-Depression	Baseline	10.0.5 (10.1 to 10.9)	9.7 (9.3 to 10.1)			
	7-week	8.9 (8.6 to 9.3)	9.1 (8.7 to 9.6)	0.9 (0.2 to 1.6)	.02	0.4 (0.1 to 0.7)
	3-month	8.8 (8.4 to 9.3)	9.2 (8.7 to 9.7)	1.0 (0.3 to 1.8)	.009	0.5 (0.1 to 0.8)
HADS-Anxiety	Baseline	9.2 8.6 to 9.8)	9.8 (9.1 to 10.5)			
	7-week	10.8 (10.2 to 11.4)	10.8 (10.2 to 11.4)	-0.6 (-1.5 to 0.4)	.25	-0.2 (-0.7 to 0.2)
	3-month	10.2 (9.5 to 10.9)	10.4 (9.7 to 11.1)	-0.3 (-1.4 to 0.7)	.51	-0.1 (-0.5 to 0.3)
Suicidality	Baseline	7.0 (4.5 to 9.5)	3.8 (1.2 to 6.3)			
	7-week	3.6 (1.0 to 6.3)	5.2 (2.3 to 8.2)	4.9 (0.9 to 8.9)	.02	0.4 (0.1 to 0.8)
	3-month	2.6 (0.1 to 5.2)	5.6 (2.8 to 8.4)	6.2 (2.3 to 10.2)	.002	0.6 (0.2 to 1.0)
GAD	Baseline	12.5 (11.3 to (13.6)	11.6 (10.4 to 12.8)			
	7-week	7.3 (6.3 to 8.4)	7.8 (6.6 to 9.0)	1.3 (-0.3 to 3.0)	.11	0.3 (-0.1 to 0.6)
	3-month	7.5 (6.3 to 8.7)	8.0 (6.7 to 9.3)	1.4 (-0.4 to 3.1)	.12	0.3 (-0.1 to 0.6)
PANAS-Positive	Baseline	20.6 (18.8 to 22.3)	21.2 (19.4 to 23.0)			
	7-week	24.5 (22.3 to 26.8)	24.2 (21.8 to 26.7)	-0.9 (-4.2 to 2.4)	.59	-0.1 (-0.5 to 0.3)
	3-month	24.5 (22.2 to 26.8)	24.3 (21.9 to 26.6)	-1.1 (-4.1 to 2.0)	.50	-0.1 (-0.1 to 0.3)
PANAS-Negative	Baseline	21.2 (19.3 to 23.1)	22.0 (20.0 to 24.0)			
	7-week	19.0 (17.0 to 21.0)	18.9 (16.8 to 21.1)	-0.9 (-4.1 to 2.4)	.60	-0.1 (-0.5 to 0.3)
	3-month	19.5 (17.5 to 21.5)	19.8 (17.6 to 22.0)	-0.5 (-3.7 to 2.6)	.74	-.0.1 (-0.4 to 0.3)
Quality of Life	Baseline	46.7 (44.4 to 49.1)	44.8 (42.4 to 47.3)			
	7-week	42.3 (39.7 to 44.9)	42.2 (39.4 to 45.0)	1.8 (-1.3 to 4.9)	.26	0.2 (-0.1 to 0.5)
	3-month	40.7 (38.0 to 43.4)	43.0 (40.0 to 46.1)	4.2 (0.5 to 7.9)	.03	0.4 (0.1 to 0.8)
COVID Concerns	Baseline	25.5 (24.0 to 27.1)	25.4 (23.8 to 27.1)			
	7-week	22.3 (20.5 to 24.2)	22.7 (20.6 to 24.7)	0.4 (-2.4 to 3.1)	.79	0.1 (-0.3 to 0.5)
	3-month	22.2 (20.3 to 24.1)	22.3 (20.1 to 24.4)	0.1 (-2.8 to 3.1)	.94	0.0 (-0.4 to 0.5)
Sleep Disturbance	Baseline	9.3 (8.4 to 10.2)	8.6 (7.6 to 9.6)			
	7-week	6.8 (5.7 o 7.8)	7.0 (5.9 to 8.1)	1.0 (-0.5 to 2.4)	.19	0.3 (-0.1 to 0.6)
	3-month	6.1 (5.0 to 7.2)	6.4 (5.0 to 7.2)	1.0 (-0.7 to 2.7)	.26	0.3 (-0.2 to 0.7)

Abbreviations. EUC = Enhanced usual care; LS = Least Square; HADS = Hospital Anxiety and Depression Scale (depression subscale score range: 0-21; anxiety subscale score range: 0-21; higher scores indicate elevated anxiety or depression); Suicidality = Suicidal Ideation Attributes Scale (total score range: 0-50; higher scores indicate more severe suicidality); GAD-7 (total score range: 0-21; higher scores indicate more severe worry); PANAS = Positive and Negative Affect Schedule (subscale total score range: 10-50 on positive and negative scales, respectively; higher scores indicate more greater positive and negative mood, respectively); Quality of Life = Australian Quality of Life scale (total score range: 20-100; higher scores indicate poorer quality of life); COVID Concerns = CSS = COVID-19 Stress Scale (each scale total score range: 0-24; higher scores indicate more severe stress); SII = Sleep Impairment Index (total score range: 0-20; higher scores indicate more severe sleep impairment). Effect size was calculated by the difference in least square means between intervention and EUC from mixed model divided by the pooled standard deviation.

Clinical Trial Protocol

Title: A Controlled Trial of Reducing COVID-19 Related Stress and Mood Problems

Short Title: Treating COVID-19 Distress

Clinical trial protocol and protocol amendment

Protocol number	Version number	Version date	
1	1	17/8/2020	

Version 1: 15/8/2020

General Information

Trial sponsor

Name	University of New South Wales
Address	UNSW Sydney, Sydney, NSW, 2052
Contact	Professor Richard Bryant: r.bryant@unsw.edu.au

Trial sponsor authorisation

Title	Name	Email
Dr	Ted Rohr	t.rohr@unsw.edu.au

Trial Sites

Site name	Site address
UNSW Sydney	UNSW Sydney Campus, Anzac Parade, Kensington, NSW, 2052

Content Expert Investigator

Title	Name	Email	Address and telephone number
Professor	Richard Bryant	r.bryant@unsw.edu.au	School of Psychology, UNSW Sydney, Sydney, NSW, 2052. Tel: 0405375874

Chief Investigator

Title	Name	Email	Address and telephone number
Professor	Richard Bryant	r.bryant@unsw.edu.au	School of Psychology, UNSW Sydney, Sydney, NSW, 2052. Tel: 0405375874

Expert to Review Adverse Events, Reactions, SUSARS, and SSI

Title	Name	Email	Address and telephone number
Professor	Richard Bryant	r.bryant@unsw.edu.au	School of Psychology, UNSW Sydney, Sydney, NSW, 2052. Tel: 0405375874

Version 1: 15/8/2020

Study Investigators

Title	Name	Email	Trial Site
Professor	Richard Bryant	r.bryant@unsw.edu.au	School of Psychology, UNSW Sydney, Sydney, NSW, 2052. Tel: 0405375874
Dr	Katie Dawson	Katie.dawson@unsw.edu.au	UNSW Sydney
Ms	Srishti Yadav	srishti.yadav@hotmail.com	UNSW Sydney
Dr	Dharani Keyan	d.keyan@unsw.edu.au	UNSW Sydney
Ms	Suzanna Azevado	suzanna.azevedo@sydney.edu.au	UNSW Sydney
Ms	Jenny Tran	jenny.tran1@unsw.edu.au	UNSW Sydney

Trial safety committees

Title	Name	Email
Professor Angela Nickerson*	UNSW Sydney	a.nickerson@unsw.edu.au
Professor Anthony Harris	University of Sydney	a.harris@unisyd.edu.au
Dr Susanne Schweizer	UNSW Sydney	s.schweizer@unsw.edu.au

*Professor Nickerson will function as the member of the Trial Safety Committee who will receive all information regarding safety reports from Professor Bryant.

Funding and resources

This trial is being funded by a NHMRC Investigator Grant (APP1173921).

Insurance

This trial is covered by the UNSW Insurer (see attached).

Trial Registration

This trial was prospectively registered on the ANZCTR on 13/8/20 (ACTRN12620000811909).

Version 1: 15/8/2020

Background information

Investigation Product
Adapted Positive Affect Training (PAT) is a 6-session psychological intervention adapted for delivery during the COVID-19 pandemic.

Summary of Evidence Pertinent to the Trial
There have been trials to reduce anxiety and depression during the pandemic. One prior trial was based on Problem Management Plus (PM+), which was developed in a collaboration between UNSW and the World Health Organization to teach people evidence-based skills to cope with stress in times of crisis[1].This program has been shown to be effective across many trials across the world in populations affected by adversity[2-5]. It includes psychoeducation, arousal reduction techniques, problem-solving skills, behavioural activation, and enhancement of social support, which are all based on the principles of cognitive-behavioural therapy. PM+ is relevant for the current situation because: (a) the strategies are simple to learn, (b) the strategies are transdiagnostic and address stress in otherwise healthy populations, and (c) can be taught in brief format. In a prior trial, PM+ was adapted for the pandemic by including elements of worry management, and it was conducted in small groups and via videoconferencing to address the needs of people in lockdown[6]. In recognition of the significant mood problems and suicidality arising from COVID-19, which can be compounded by the reduction of rewarding experiences during lockdowns, the a program has been adapted to focus on elements that specifically anhedonia. Specifically, this program adapts elements from Positive Affect Training, which includes positive event scheduling and imagination exercises to refine hedonic capacity[7]. This program has been shown in randomized controlled trials to decrease stress levels, improve mood, and decrease suicidal risk[8].

Potential Risks and Benefits
Across numerous large trials involving hundreds of participants, there have been no risks reported. The primary benefit is improved mental health, evidenced by reduced anxiety, depression, and posttraumatic stress.

Description and Justification for Administration and Dosage
Positive Affect Training (PAT) as used in this trial is based on cognitive behavioural strategies that have been shown to reduce stress. It is delivered in small groups across 6 sessions, and teaches participants strategies in education about stress, and then focuses strategies to boost awareness of positive experiences, including planning positive events, imagination exercises to savour the positive affect experienced when doing them, and reviewing each acitivty[8]. Sessions are typically 60 minutes duration and occur on a once-weekly basis. The PAT protocol will be administered in a small group format, which we have shown is effective in prior controlled trials. In the context of social distancing and quarantine rules, this program will be delivered via videoonferencing platforms. PAT will be administered by a Masters or Doctoral level clinical psychologist. Administration will be in

Bryant R, *et al. BMJ Ment Health* 2023; 26:e300737. doi: 10.1136/bmjment-2023-300737

small groups (N = 4) at a time. The program is based on 6 x 60-minute teleconference sessions on a once-weekly basis.

Adherence to Protocol
This trial will comply with all procedures in the Trial Protocol and will adhere to the ICH Good Clinical Practice Guidelines.

Population to Be Studied
Participants in this trial will be adults (>18years) who are reporting psychological distress related to COVID-19. This population is essential for testing this program because this program was specifically designed to reduce COVID-19 related distress, and accordingly it needs to be tested on a population psychologically affected by issues surrounding COVID-19. Distress is defined as scoring ≥16 on the K10, a psychometrically strong instrument to measure common mental disorders.

Background to the Trial
The global impact of COVID-19 has been profound, and the public health threat it represents is the most serious seen in recent pandemic history. Thus, concerns about COVID-19 are pervasive and have several serious adverse impacts on hospital systems, health workers, and the general community. A recent rapid review published in *The Lancet* in the context of COVID-19 found that studies of people forced into quarantine suffer markedly worse mental health and subsequent stress-related problems that those not quarantined[9] . For example, one study found that those quarantined during the SARS outbreak suffered worse acute stress reactions than those not guarantined[10]; these people were also more likely to report exhaustion, insomnia, irritability, poor work performance, or concentration, and reluctance to attend work. A further study found that quarantine in health workers resulted in markedly higher rates of depression[11]. The longer the quarantine, the worse the mental health[12]. These stress reactions are associated with fear of infection or infecting others[13], financial stressors[14], and inadequate supplies[15]. In summary, the stressors placed upon people, including health workers, during a pandemic are likely to negatively impact one's mental health.

There is an urgent demand for programs that can address the stress experienced by people during the COVID-19 pandemic. This situation does not require therapy because these personnel do not necessarily suffer from a mental disorder. Instead, there are huge numbers of people who are facing unprecedented levels of stress and require strategies to manage this stress.

Trial Objectives and Purpose
This study will test the capacity of an adapted version of PAT to reduce distress associated with COVID-19. In light of the need for social distancing and quarantine, it will deliver this via teleconferencing and on a small group basis. The trial will compare PAT with provision of comparable resources that outline the coping strategies but the participant reviews the strategies in a self-guided manner. This study aims to test whether the adapted version of

Bryant R, *et al. BMJ Ment Health* 2023; 26:e300737. doi: 10.1136/bmjment-2023-300737

PAT specifically designed to target COVID-19 related distress, and mood problem and suicidality, will result in reduce distress relative to usual care.

Trial design

This is a single-blind, parallel, randomized controlled trial. The project will employ a 2 (Treatment Condition) x 4 (Assessment Point) factorial design. That is, participants will be randomly allocated to either (a) Positive Affect Training (PAT), or (b) Enhanced Usual Care (EUC). All participants will be assessed (a) pre-treatment, (b) post-treatment, (c) 3-months post-treatment. The primary outcome timepoint will be the 3-months assessment; this is done to prioritise rapid implementation of the findings in the context of the current COVID-19 crisis.

Investigational product dispensing and packaging

Not applicable to PAT. Clinical psychologists will follow the PAT protocol and deliver the content via teleconferencing.

Trial Duration

Each participant will be involved in the trial for a total period of 20 weeks; this comprises the baseline assessment, 6 weeks of PAT or control, post-assessment, and 3-month assessment.

It is expected that the trial will follow the following timelines:

Participant recruitment:	September – December 2020
Baseline assessment:	September – December 2020
Intervention:	September – December 2020
Post-assessment:	October 2020- January 2021
Three-month assessment:	January 2021-April 2021

Trial Documentation

**Documentations**	**Version #**	**Version date**	**Appendix #**
Invitation email	**NA**		
Study advertisement	**1**	**15/8/20**	**1**
Participant information sheet and consent form	**1**	**15/8/20**	**2**
Screening data collection tool	**1**	**15/8/20**	**3**
Data collection tool	**1**	**15/8/20**	**4**
Safety monitoring report form	**1**	**15/8/20**	**5**
Clinical Trial Notification receipt	**NA**	**-**	**-**
Insurance Proof	**1**	**15/8/20**	**6**
Trial Registration	**1**	**15/8/20**	**7**

Study treatments and procedures

Version 1: 15/8/2020

The proposed study will follow CONSORT recommendations for conduct and reporting of randomised controlled trials (Schulz, Altman & Moher, 2010) including (a) clearly defined target symptoms, measured both pre and post intervention, (b) standardised and reliable assessment procedure, (c) blind assessments, (d) standard training of assessors and inter-rater reliability checks to ensure standardisation, (e) manualised and replicable treatment procedures, (f) random allocation to treatment, and (g) treatment fidelity checks to ensure treatment adherence.

Procedure

Recruitment and Initial Assessment:

Participants will be people who contact the UNSW Traumatic Stress Clinic to participate in the program. The program will be advertised on the UNSW Traumatic Stress Clinic website. Participants identified as satisfying inclusion criteria if they score ≥16 on the K10[16]. Following explanation of the trial, participants will complete online informed consent procedures. Participants will then complete a baseline assessment online that will take approximately 30 minutes.

Following explanation of the trial, participants will complete online informed consent procedures. Participants will then complete a baseline assessment online that will take approximately 30 minutes (measures listed below).

Randomization

Randomisation will be conducted following the initial assessment by a computerized program and administered by personnel at UNSW who are independent of this treatment study. Randomisation will occur on a 1:1 basis. Assessments will be managed by a psychologist who is independent of treatment condition.

Introductory Online Session

Participants randomised to PAT will be asked to participate in an initial 10 minute teleconference interview with their group facilitator for introductory purposes, explain technological issues involved with the Doxy platform, and explain in basic terms how the program will operate.

Treatment

Participants will receive 6 x 60-minute sessions provided on a weekly basis by a clinical psychologist at the UNSW Traumatic Stress Clinic. Treatment will be delivered via teleconference to 4 people at a time.

PAT

PAT components are based on evidence-based strategies to reduce stress. This program will include:

(a) Education about stress
(b) Positive experience strategies
(d) Relapse prevention

Version 1: 15/8/2020

Education will provide education abut stress reactions to COVID-19.
Positive experience strategies will introduce strategies regarding structured scheduling of daily exposure to positive experiences, including directive rehearsal of imagination exercises to practice awareness of positive aspects of this activity
Relapse prevention will involve teaching participants relapse prevention techniques, including identification of possible future relapse situations and rehearsal of strategies to manage these situations.

Control Arm
Participants randomized to the Control condition will be emailed pdf handouts of the strategies comparable to the ones taught in the PAT arm. Participants will complete these in a self-guided and self-paced manner.

Post-Treatment Assessment:

Patients will complete an assessment immediately following the treatment intervention by completing an online survey. Participants will also be asked to complete the same questionnaires regarding distress and coping that were completed at Baseline.

Three Month Follow-Up Assessment

Three months after the treatment intervention, participants will complete a follow-up assessment by completing an online survey. Participants will also be asked to complete the same questionnaires regarding distress and coping that were completed at Baseline. Participants who complete the 3-month assessment will be reimbursed $50 for their time.

Version 1: 15/8/2020

Study Flow Chart

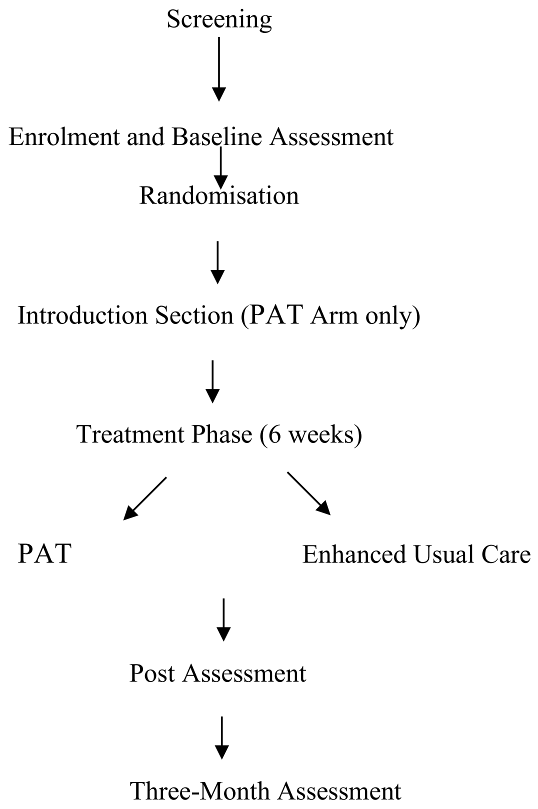

Version 1: 15/8/2020

Outline of Session Contents

List Interventions	Enrolment Session	Sessions 1-6	Session 7	Session 8	Final Session
Informed Consent	✓				
Inclusion / Exclusion critieria	✓				
PAT sessions		✓			
Assessment	✓		✓	✓	✓
Adverse Event & Serious Adverse Event Assessment	✓	✓	✓	✓	✓

Contraindications

The only contraindication for this trial will be concurrent treatment by a clinical psychologist.

Subject compliance

Treatment Checks.

Each therapist will be trained in the respective therapy techniques by Bryant, and will follow a detailed treatment manual. All sessions will be audiotaped and fidelity checks of 10% of sessions for each treatment condition will be independently rated by independent experts. There will be16 specific components to be rated for PAT. The independent experts will be Masters or Doctoral trained Clinical Psychologists. To ease the burden of rating of many treatment sessions, different raters will be recruited throughout the course of the trial. Sessions will not be transcribed but raters will make ratings on the basis of audiofiles that can be accessed by encrypted passwords. Each Clinical Psychologist will sign a confidentiality agreement indicating that they will not disclose any information from the sessions they rate. Raters will be blind to treatment condition of the sessions they rate, and will be required to nominate what therapy components were present in each session and the quality with which these components were administered. The CI's previous studies have used these fidelity checks to ensure optimal indexing of the integrity of the treatment conditions. This system will ensure the validity and standardisation of the respective treatment conditions

Ethics

Human research ethics approval is currently under review.

HREC Name	Ethics Reference	Ethics approval date	Ethics expiration date
UNSW HREC			

Version 1: 15/8/2020

Selection and Withdrawal

Recruitment

The Traumatic Stress Clinic will receive participants who can access information about the trial from the Traumatic Stress Clinic portal (http://www.traumaticstressclinic.com.au/).The investigators will make no direct contact with potential participants prior to their approach to the clinic. Individuals who are interested in taking part in the study will read an explanation of the trial and then provide informed consent. They will then be directed to a webpage that that will include the K10; if participants score ≥16 on the K10 they will be randomised to either PAT or Control.

Eligibility Criteria

Inclusion Criteria:

- Score of ≥16 on the K10
- Aged at least 18 years
- Sufficient English language comprehension
- Access to videoconferencing platform

Screening

Exclusion Criteria:

- Current psychosis
- Imminent suicidal risk
- Current substance dependence (but not abuse)
- No access to internet-based access to teleconferencing facility

Withdrawal of study participants

The following criteria will be applied for withdrawal of study participants:

a) Participants will be withdrawn from the study if they display marked deterioration in mental health during the program or follow-up period. In such cases the clinical psychologist responsible for this case will offer the participant a referral to an appropriate mental health specialist
b) All participants who are withdrawn from the study for clinical reasons will be retained in the analyses. If participants wish to withdraw from the study, all their data will be removed from the database.
c) Participants will not be replaced. Outcome analyses are based on intent-to-treat analyses.
d) All participants will be followed up, including those who drop out of treatment or the clinical psychologist deems should not proceed with the program, at the designated times. Only participants who express the desire to withdraw from the study will not be followed up.

Treatment of Participants

1. Participants in the PAT arm will be receive 6 x 1-hour videoconferencing sessions occurring on a weekly basis, delivered by a Masters/Doctoral level psychologist to groups of 4 people.
2. Participants in the EUC arm will be emailed pdf handouts of the strategies comparable to the ones taught in the PAT arm. Participants will complete these in a self-guided and self-paced manner.

Version 1: 15/8/2020

3. Concurrent psychotropic medications are permitted in this trial but will be recorded on assessment.
4. Compliance with PAT be monitored by recording attendance at each videoconference session.

Assessment of Efficacy

Efficacy will be assessed by conducting independent assessments via online questionnaires at baseline, immediately after the 6th session, and 3-months post-treatment. The primary outcome time point will be the 3-month assessment.

Measures

The assessment measures used in the study are directed by the prevailing literature on common mental disorders.

Self-Report Measures

- The primary outcome measured will be severity of anxiety and depressive symptoms measured using the Hospital Anxiety and Depression Scales (HADS[17]). The HADS is a 14-item scale consisting of 2 sub-scales: HADS-A (anxiety, 7 items, range 0-21) and HADS-D (depression, 7 items, range 0-21). Higher scores indicate more anxiety and/or depression. The minimal clinically important difference has been determined at 1.32 for HADS-A and 1.40 for HADS-D.
- Generalized Anxiety Disorder Scale (GAD-7[18]) is a 7-item measure that was designed to assess for generalized anxiety scale. It is focused on worry items, and so relevant to concerns about COIVD-19. It possess good reliability, as well as criterion, construct, factorial, and procedural validity. A cut point was identified that optimized sensitivity (89%) and specificity (82%).
- Sleep Impairment Index (SII[19]) was used to assess sleep difficulties. The SII is a 7-item measure of sleep impairment; this study used 5 of the 7 items because of time constraints within a larger clinical interview. The items indexed impairment in sleep onset, maintenance, early waking, disturbance caused by sleep problems, and distress caused by sleep problems (this version omitted items indexing satisfaction with sleep and the extent to which sleep problems were noticeable by others). The internal consistency of the 5-item version was 0.87, and the item-total correlations varied from 0.46 (waking early) to 1.00 (delayed onset sleep), with a mean correlation of 0.57. These values are similar to the values reported for the 7-item measures[16] , and validate our use of the 5-item scale. Each item is rated on 5-point rating scale (0-4) providing a potential total score of 28, and the recommended cut-off for identifying insomnia is 14. Accordingly, in recognition that we used 5 of the 7 items, we adopted a pro-rated score of 12 as a conservative index of sleep disturbance.
- Suicide risk will be assessed by the Scale for Suicidal Ideation (SSI[20]), which is a 19-item questionnaire in which each item is scored on 3-point scale, providing a range of 0-38. It measures that indexes active suicidal desire, passive suicidal desire, and plans for suicide.
- COVID Concerns Scale measures is a scale developed for this study that indexes the extent the participant engages in worry about COVID-19 related issues.
  Positive and negative affect will be assessed with the Positive and Negative Affect Schedule (PANAS[21]) on which participants describe their mood by rating 10 positive and 10 negative words.
- Health related quality of life will be measured using Australian Quality of Life (AQoL-8D[22]). This measures quality of life and health outcomes across eight domains

Version 1: 15/8/2020

(independent living, relationships, mental health, coping, pain, senses, self-worth and happiness) and will be used to calculate quality adjusted life years (QALYs) for the economic evaluation.

Assessment of Safety

There are no expected adverse reactions from this study. This intervention is based on a psychological program that teaches simple coping strategies with stress. PM+ has been trialed in three major controlled trials, and across 700 participants there was not a single adverse event arising from the PM+ intervention. PAT has also been trialed across multiple trials with no serious adverse events. Further, any participants who are assessed as being at risk of suicide will be excluded from the study prior to randomization and they will be referred to local services. This results in the trial posing minimum risk to participants.

**Adverse Event (AE)**	Any untoward medical or psychological occurrence in a participant that is not related to the PAT intervention, including contracting COVID-19, will be reported in the study on a standard form and submitted to the Chief Investigator, Prof R. Bryant within 48 hours. It will also be summarised in a Safety Monitoring Register, and submitted to the Trial Safety Committee (specifically to Prof Angela Nickerson) and Trial Sponsor on a 6-monthly basis.
**Adverse Reaction (AR)**	Any untoward medical or psychological occurrence in a participant that is related to the PAT intervention, including contracting COVID-19, will be reported in the study on a standard form and submitted to the Chief Investigator, Prof R. Bryant within 48 hours. It will also be summarised in a Safety Monitoring Register, and submitted to the Trial Safety Committee (specifically to Prof Angela Nickerson) and Trial Sponsor on a 6-monthly basis.
**Serious Adverse Event (SAE)/Serious Adverse Reaction (SAR)**	Any adverse event/adverse reaction that results in death, is life-threatening, requires hospitalisation or prolongation of existing hospitalisation, results in persistent or significant disability or incapacity, or is a congenital anomaly or birth defect.<br>**Reporting**<br>• Any serious adverse events will be reported via email to the Trial Sponsor .
**Significant Safety Issue (SSI)**	Across multiple controlled trials of PAT, there have not been direct safety events reported as a result of the intervention. This is understandable in light of the content of PAT being a psychological strategy to teach coping skills. However, if any unforeseen SSI occurs that suggests that PAT does represent a safety issue for any participants that will be emailed within 72 hours to the Trial Sponsor .
**Suspected Unexpected Serious Adverse Reaction (SUSAR)**	• The nature of PAT is that it cannot be the cause of SUSARs. However, any cases in the study who do suffer death (potentially as a result of COVID-19) will be reported to the Trial Sponsor.

Statistics

Power analysis indicated a minimum sample size of 67 participants per group power = 0.95, alpha = 0.05, two-sided) to achieve an effect size of 0.5 between condition; allowing for attrition of 30% at follow-up, this study needs to recruit 174 participants.

Version 1: 15/8/2020

Analyses will focus primarily on intent-to-treat analysis. Hierarchical linear mixed models (HLM) will be used to study differential effects of each treatment condition because this method effectively handles missing data by calculating estimates of trajectories. For the posttreatment analyses between the two conditions, analyses will focus on linear time effects, treatment conditions, and interactions. Fixed effects parameters were tested with the Wald test (t-test, p <.05, two-sided) and 95% confidence intervals. Cohen's (d) effect size was calculated for all analyses. The primary outcome measure will be the HADS. The primary outcome timepoint will be the 3 months assessment. Given the speed of this trial, there will be no interim analyses to determine if the trial needs to be terminated prematurely.

Direct Access to Source Data/Documents

Annual audits will be conducted by UNSW HREC and reports will be provided by the Chief Investigator to HREC.

The database will be available for regular monitoring.

Quality Control

Implementation of the trial protocol will be monitored on a weekly basis by the Chief Investigator.

Ethics

As outlined above, this trial will comply with the approval outlined by the UNSW Human Research Ethics Committee. Informed consent will be obtained, all participants have the right to withdraw and withdraw their data, and any adverse events arising will be reported and appropriate actions taken to ensure the well-being of the participants.

Data Handling

As this is a treatment trial, participants will be required to have their identities known to the study because they are seen on a weekly basis, and then be contacted for follow-up assessment. All data entered by participants during the online surveys collected at pretreatment, posttreament, and follow-up will be entered on an electronic database in a deidentified manner in which participants are allocated a unique identification number. This information is linked to the participant's information in a separate file that is locked in secure filing cabinets in the Mathews Building (Room 1118). Electronic files will be stored on a secure IT network accessible only to staff directly involved in the study. Access will be restricted to research staff directly involved in the study. If the data is moved to any other computer location it will have AES-256 bit encryption. We routinely audiorecord therapy sessions in line with CONSORT requirements for subsequent testing of treatment fidelity via independent coding of experts. The research staff working on the project will initially have access to the data via the UNSW Research Long Term Data Store Interface.

Publication Policy

Version 1: 15/8/2020

The results from this project will be communicated to the public in the form of peer-reviewed journals; where such journals are not open-access, the articles will be made available through the institutional repository UNSWorks. All publications will ensure that all results report aggregated data that contains no identifiable data of individuals.

References

1. Dawson KS, Bryant RA, Harper M, et al. Problem Management Plus (PM+): a WHO transdiagnostic psychological intervention for common mental health problems. *World Psychiatry* 2015; **14**(3): 354-7.
2. Rahman A, Hamdani SU, Awan NR, et al. Effect of a Multicomponent Behavioral Intervention in Adults Impaired by Psychological Distress in a Conflict-Affected Area of Pakistan: A Randomized Clinical Trial. *JAMA* 2016; **316**(24): 2609-17.
3. Bryant RA, Schafer A, Dawson KS, et al. Effectiveness of a brief behavioural intervention on psychological distress among women with a history of gender-based violence in urban Kenya: A randomised clinical trial. *PLoS Med* 2017; **14**(8): e1002371.
4. Bryant RA, Bawaneh, A., Awwad, M., Al-Hayek, H., Giardinelli, L., Whitney, C., Jordans, M.J.D., Cuijpers, P., Sijbrandij, M., Ventevogel, P., Dawson, K., & Akhtar, A. Effectiveness of a brief group behavioral intervention for common mental disorders in Syrian refugees in Jordan: A randomized controlled trial. *PLoS Medicine* 2022; **19**(3): e1003949.
5. Rahman A, Khan, M.N., Hamdani, S.U., Chiumento, A., Akhtar, P., Nazir, H., Nisar, A., Masood, A., Din, I.U., Khan, N.A., Bryant, R.A., Dawson, K.S., Sijbrandij, M., Wang, D., & van Ommeren, M. Effectiveness of a brief group psychological intervention for women in a post-conflict setting in Pakistan: a cluster randomized controlled trial. *Lancet* 2019; **393**: 1733-44.
6. Bryant RA, Dawson KS, Keyan D, et al. Effectiveness of a Videoconferencing-Delivered Psychological Intervention for Mental Health Problems during COVID-19: A Proof-of-Concept Randomized Clinical Trial. *Psychother Psychosom* 2021: 1-10.
7. Craske MG, Meuret AE, Ritz T, Treanor M, Dour HJ. Treatment for Anhedonia: A Neuroscience Driven Approach. *Depress Anxiety* 2016; **33**(10): 927-38.
8. Craske MG, Meuret AE, Ritz T, Treanor M, Dour H, Rosenfield D. Positive affect treatment for depression and anxiety: A randomized clinical trial for a core feature of anhedonia. *J Consult Clin Psychol* 2019; **87**(5): 457-71.
9. Brooks SK, Webster RK, Smith LE, et al. The psychological impact of quarantine and how to reduce it: rapid review of the evidence. *Lancet* 2020; **395**(10227): 912-20.
10. Bai Y, Lin CC, Lin CY, Chen JY, Chue CM, Chou P. Survey of stress reactions among health care workers involved with the SARS outbreak. *Psychiatr Serv* 2004; **55**(9): 1055-7.
11. Liu X, Kakade M, Fuller CJ, et al. Depression after exposure to stressful events: lessons learned from the severe acute respiratory syndrome epidemic. *Compr Psychiatry* 2012; **53**(1): 15-23.
12. Hawryluck L, Gold WL, Robinson S, Pogorski S, Galea S, Styra R. SARS control and psychological effects of quarantine, Toronto, Canada. *Emerging Infectious Diseases* 2004; **10**(7): 1206-12.
13. Reynolds DL, Garay JR, Deamond SL, Moran MK, Gold W, Styra R. Understanding, compliance and psychological impact of the SARS quarantine experience. *Epidemiol Infect* 2008; **136**(7): 997-1007.

Version 1: 15/8/2020

14. Cava MA, Fay KE, Beanlands HJ, McCay EA, Wignall R. The experience of quarantine for individuals affected by SARS in Toronto. *Public Health Nurs* 2005; **22**(5): 398-406.
15. Desclaux A, Badji D, Ndione AG, Sow K. Accepted monitoring or endured quarantine? Ebola contacts' perceptions in Senegal. *Soc Sci Med* 2017; **178**: 38-45.
16. Kessler RC, Andrews G, Colpe LJ, et al. Short screening scales to monitor population prevalences and trends in non-specific psychological distress. *Psychological Medicine* 2002; **32**(6): 959-76.
17. Zigmond A, Snaith R. The Hospital Anxiety and Depression Scale. *Acta Psychiatrica Scandinavia* 1983; **67**: 361-70.
18. Spitzer RL, Kroenke K, Williams JB, Lowe B. A brief measure for assessing generalized anxiety disorder: the GAD-7. *Arch Intern Med* 2006; **166**(10): 1092-7.
19. Morin C, Stone, J, McDonald, K, Jones, S. Psychological management of insomnia: a clinical replication series with 100 patients. *Behavior Therapy* 1994; **25**: 291-309.
20. van Spijker BA, Batterham PJ, Calear AL, et al. The suicidal ideation attributes scale (SIDAS): Community-based validation study of a new scale for the measurement of suicidal ideation. *Suicide Life Threat Behav* 2014; **44**(4): 408-19.
21. Watson D, Clark LA, Tellegen A. Development and validation of brief measures of positive and negative affect: the PANAS scales. *J Pers Soc Psychol* 1988; **54**(6): 1063-70.
22. Richardson JR, Iezzi, A., Khan., M.A., & Maxwell, A. Validity and reliability of the Assessment of Quality of Life (AQoL)-8D Multi-Attribute Utility Instrument. *The Patient - Patient Centered Outcomes Research* 2014; **7**: 85-96.

Version 1: 15/8/2020
